# Pharmacogenetic and pharmacokinetic factors for dexmedetomidine-associated hemodynamic instability in pediatric patients

**DOI:** 10.3389/fphar.2024.1515523

**Published:** 2025-01-07

**Authors:** Yanping Guan, Bilian Li, Yiyu Zhang, Hao Luo, Xueding Wang, Xue Bai, Zhuoling Zheng, Yaying Huang, Wei Wei, Min Huang, Xingrong Song, Guoping Zhong

**Affiliations:** ^1^ Institute of Clinical Pharmacology, School of Pharmaceutical Sciences, Sun Yat-Sen University, Guangzhou, Guangdong Province, China; ^2^ Department of Anesthesiology, Guangzhou Women and Children’s Medical Center, Guangzhou Medical University, Guangzhou, Guangdong Province, China; ^3^ Department of Pharmacy, Sun Yat-sen University Sixth Affiliated Hospital, Sun Yat-Sen University, Guangzhou, Guangdong Province, China

**Keywords:** dexmedetomidine, hemodynamic instability, single-nucleotide polymorphism, pharmacokinetics, pediatric anesthesia

## Abstract

**Purpose:**

The incidence of hemodynamic instability associated with dexmedetomidine (DEX) sedation has been reported to exceed 50%, with substantial inter-individual variability in response. Genetic factors have been suggested to contribute significantly to such variation. The aim of this study was to identify the clinical, pharmacokinetic, and genetic factors associated with DEX-induced hemodynamic instability in pediatric anesthesia patients.

**Methods:**

A cohort of 270 pediatric patients scheduled for elective interventional surgery received an intranasal dose of 3 mcg·kg^-1^ of dexmedetomidine, and subsequent propofol induction was conducted when patients had a UMSS of 2–4. The primary endpoint was hemodynamic instability—defined as a composite of hypotension and/or bradycardia, which is characterized by a 20% reduction from age-specific baseline values. Plasma concentrations of dexmedetomidine were determined, and single-nucleotide polymorphisms (SNPs) were genotyped. A validated population pharmacokinetic model was used to estimate pharmacokinetic parameters. LASSO regression was used to identify significant factors, and a Cox’s proportional hazards model-derived nomogram for hemodynamic instability was developed.

**Results:**

Hemodynamic instability was observed in 52 out of 270 patients (209 events), resulting in a cumulative incidence of 16.30% at 90 min, as estimated by Kaplan–Meier estimation, and it was associated with a median time to event of 35 min. The interval time between DEX initiation and propofol induction was 16 min (IQR: 12–22 min). The cumulative incidence was 8.2% within 22 min after DEX initiation. The identified significant risk factors for DEX-associated hemodynamic instability included weight, DEX clearance, concomitant propofol use, and the following gene variants *UGT2B10* rs1841042 (hazard ratio (HR):1.41, 95% confidence interval (CI): 1.12–1.79), *CYP2A6* rs8192733 (HR:0.28, 95%CI:0.09–0.88), *ADRA2B* rs3813662 (HR:1.39,95%CI:1.02–1.89), *CACNA2D2* rs2236957 (HR:1.46, 95%CI:1.09–1.96), *NR1I2* rs3814057 (HR:0.64, 95%CI:0.43–0.95), and *CACNB2* rs10764319 (HR:1.40,95%CI:1.05–1.87). The areas under the curve for the training and test cohorts were 0.881 and 0.762, respectively. The calibration curve indicated excellent agreement.

**Conclusion:**

The predictive nomogram, which incorporates genetic variants (*UGT2B10, CYP2A6, ADRA2B, CACNA2D2, NR1I2*, and *CACNB2*) along with clinical factors such as weight, DEX clearance, and propofol use, may help prevent DEX-associated hemodynamic instability. Delayed hemodynamic instability is likely to occur after 35-min DEX initiation in patients with lower DEX clearance after propofol induction.

## 1 Introduction

Dexmedetomidine (DEX), a popular anesthetic, has been widely used as an anesthetic adjunct to pediatric anesthesia. It has a short half-life, promotes a calm emergence, and is associated with the maintenance of airway stability and spontaneous ventilation (Mason and Lerman, 2011). Although DEX may be more effective and less harmful than other anesthetics, it has led to adverse effects such as hypotension and bradycardia, which are attributable to its α2 agonist activity (Mahmoud and Mason, 2015). Such hemodynamic instability is highlighted by its high incidence with obvious inter-individual differences (Okello et al., 2018) and the need for corrective interventions. However, despite their clinical significance, the occurrence of such hemodynamic instability is generally under-reported and not well-studied (Mason and Lerman, 2011).

DEX exerts its pharmacological effects primarily through selective activation of the α2-adrenergic receptors (α2-AR), particularly within the locus coeruleus (Scheinin and Schwinn, 1992). This action results in anti-sympathetic, sedative, anxiolytic, and analgesic effects without causing respiratory depression (Ingrande and Lemmens, 2013). It occupies a_2A-_, a_2B-_, and a_2C-_ receptors, leading to different pharmacological effects (Belfer et al., 2005; Bulow et al., 2016). Several molecular pathways have been implicated in a2-AR-mediated cellular signaling. a_2_-ARs can increase the activity of protein kinase C (PKC) through Gq-type G-proteins, which has been reported to contribute toward membrane depolarization, Ca^2+^ influx, and smooth muscle contraction (Parkinson and Hughes, 1995; Gesek, 1996). Interaction of a_2_-ARs with voltage-sensitive Ca^2+^ channels has been shown to elicit extracellular Ca^2+^ influx (Parkinson and Hughes, 1995), and these calcium channels can act both on the cardiac tissue and vascular smooth muscles. These calcium channels influence both cardiac tissue and vascular smooth muscle, suggesting that genetic polymorphisms in α2-ARs, PKC, or calcium channels may contribute to the hemodynamic fluctuations observed following DEX administration.

Plasma DEX concentrations have been shown to correlate with the sedation levels (Holliday et al., 2014), with variations in drug absorption, distribution, metabolism, and elimination that could be attributed to efficacy and toxicity (Zheng et al., 2004). DEX is predominantly metabolized in the liver by cytochrome P450 enzyme CYP2A6 and by UDP-glucuronosyltransferases (UGTs) such as UGT1A4 and UGT2B10 (Kaivosaari et al., 2008; Su and Hammer, 2011). Genetic polymorphisms in CYP2A6 expression have been well-documented, and such variations can lead to significant inter-individual differences in the pharmacokinetics of DEX (Raunio et al., 2001; Carter et al., 2004). Furthermore, population-based studies have reported differences in the frequency of CYP2A6 alleles across Asian, European, and Caucasian populations, further complicating the pharmacokinetics of DEX. In terms of phase-II metabolism, polymorphisms in UGT enzymes have also been implicated in variations in drug metabolism (Raunio et al., 2001; Chatzistefanidis et al., 2012). However, the roles of drug metabolizing enzymes and drug exposure in hemodynamic fluctuation are still being elucidated.

Identification of factors related to DEX-associated hemodynamic instability can help determine which patients could benefit from adjunct DEX treatment. Accordingly, we hypothesized that the genetic variants would be associated with the development of hemodynamic instability. The aim of the study was to characterize the candidate SNPs in the drug target (a_2_-ARs), a_2_-AR-mediated cellular signaling (PKC and calcium channels), and metabolizing enzymes (CYP2A6 and UGTs) and determine the variable factors in a relatively large cohort of infants and children. In addition, we also aimed to identify pharmacokinetic factors associated with the hemodynamic instability. Finally, our goal was to develop and externally validate a predictive model to aid in clinical decision-making for this patient population.

## 2 Methods

### 2.1 Study design

This study was approved by the Ethics Committee of Guangzhou Women and Children’s Medical Center (2017121406), and the trial was registered prior to patient enrollment at the Chinese Clinical Trial Registry (http://chictr.org.cn,ChiCTR1800015340, principal investigator: Bi Lian Li, date of registration: 2018/03/24). Written informed consent was obtained from all patients’ legal guardians or parents before the study.

### 2.2 Inclusion and exclusion criteria

Children undergoing interventional therapy, aged between 3 and 72 months, requiring general anesthesia were enrolled in this clinical trial. The inclusion criteria included adequate hematological, hepatic, and renal functions (National Cancer Institute, 2017); no consumption of DEX or any other sedative within a week before surgery; and classification as class II and III on the American Society of Anesthesiologists (ASA) scale. The exclusion criteria were as follows: 1) patients with neurological disorders; 2) organ dysfunction; 3) significant developmental delays or behavior problems; 4) allergy or hypersensitive reaction to DEX; 5) current treatment with α-adrenergic, β-adrenergic agonists, antagonists, or enzyme inducers or enzyme inhibitors of DEX; 6) the presence of active respiratory symptoms or rhinorrhea that might influence nasal drug absorption.

### 2.3 Clinical protocol

DEX at 3 mcg·kg^−1^ (Ai Bei Ning; Jiang Su Heng Rui Medicine Co. Ltd.) was administered via atomization using the Mucosal Atomization Device (Teleflex MAD Nasal; Research Triangle Park) 30–40 min before propofol induction. The target UMSS levels (University of Michigan Sedation Scale, UMSS, Supplementary Table S1) (Malviya et al., 2002) were 2–4 after DEX initiation before propofol induction. The time interval between dexmedetomidine administration and propofol induction was recorded. Subsequently, all patients received 2 mg·kg^−1^ propofol, 0.3 mcg·kg^−1^ sufentanil, and 0.2 mg·kg^−1^ cisatracurium besylate and had laryngeal mask airway placement or tracheal intubation. General anesthesia was maintained with sevoflurane inhalation (0.5%–3%). Medications administered before and during the study period were recorded. Significant signs (changeover 20% from the baseline values) were recorded every 15 min after the completion of the operation until the patient was awake and ready to be discharged.

We hypothesized that the individual variations in DEX-induced hemodynamic instability could be partially explained by clinical characteristics, DEX pharmacokinetics, and pharmacogenetics. The primary outcome was hemodynamic instability, defined as a composite of hypotension and/or bradycardia, with hypotension being a 20% reduction from age-related baseline values in systolic or diastolic blood pressure and bradycardia being a 20% reduction from age-related baseline values in heart rate (Yuen et al., 2008). In cases of hypotension or bradycardia during the procedure, intravenous epinephrine (40–100 μg) or dopamine (<3 μg kg·min^−1^) was administered. Successful sedation was defined as achieving a UMSS score of 2–4 within 30 min, as assessed by an experienced pediatric anesthesiologist. Hypoxia was defined as SpO_2_ ≤ 93% or ≥5% decrease from baseline. These identified events were used to assess the association between hemodynamic instability and the collected factors.

### 2.4 Plasma dexmedetomidine and pharmacokinetic assessment

Two milliliters of blood were collected at baseline and 60 min after DEX administration from each patient. Plasma DEX was measured by a validated high-performance liquid chromatography/tandem mass spectrometry (LC–MS/MS) method (Guan et al., 2019). In brief, sample preparation was performed by liquid–liquid extraction. Plasma aliquots of 100 μL were mixed with a stable isotope-labeled internal standard (dexmedetomidine-D_3_) and extracted with 500 μL ethyl acetate. After 3 min of vortex mixing and 10 min of centrifugation (4°C, 15,000 rpm), the supernatant was evaporated to dryness and the residues were reconstituted in the mobile phase. Isocratic HPLC separation was performed with an ACQUITY BEH C18 column (2.1 mm × 50 mm, 1.7 μm particle size; Waters, United States) and a gradient mobile phase consisting of acetonitrile and 1‰ formic acid in water (flow rate 0.3 mL·min^−1^) at 40°C. Mass spectrometric detection was carried out with a TSQ Quantum triple-quadrupole instrument using a positive selected reaction mode. The precursor–fragment ion pairs detected were m/z 201.3 → 95.1 for DEX and m/z 204.2 →98.0 for the internal standard. Mass parameters were 4,000 V ion spray voltage, 20 psi sheath gas pressure, 280°C vaporizer temperature, 5 psi auxiliary gas pressure, and 350°C capillary temperature. The lower limit of reliable quantitation of the method was 0.05 ng·mL^−1^, and the linear range was 0.05–10 ng·mL^−1^ with a correlation coefficient ≥0.99. The within- and between-run accuracy and precision of the bioassay (coefficient of variation) was within ±15% in the relevant concentration range. No significant carry-over and matrix effects were observed.

The pharmacokinetic parameters for each individual were estimated using a Bayesian approach and the *post hoc* analysis. The pharmacokinetic parameters and residual variability were assessed based on a previous pharmacokinetic study of intranasal DEX (Li et al., 2022). The population pharmacokinetic model served as *a priori* information for Bayesian forecasting, utilizing a limited sampling strategy. The apparent clearance (CL/F, Q/F) and volume of distribution (V/F, V_2_/F) for the population pharmacokinetic model are described in Equations 1–4:

$$CL/F=53.0\;*\;{\left(\frac{Weight}{70}\right)}^{0.75}*\left(\frac{{PMA}^{3.0}}{{PMA}^{3.0}+44.7^{3.0}}\right),$$ (1)

$$V/F=57.5*\left(\frac{Weight}{70}\right),$$ (2)

$$Q/F=243.8*{\left(\frac{Weight}{70}\right)}^{0.75},$$ (3)

$$V_{2}/F=71.8*\left(\frac{Weight}{70}\right),$$ (4)

where CL/F is apparent clearance of the central compartment, V/F is the central volume of distribution, Q/F is apparent clearance of the peripheral compartment, V_2_/F is the peripheral volume of distribution, weight is the patient’s body weight, and PMA is the postmenstrual age.

The individual plasma concentrations–time profiles were estimated to calculate pharmacokinetic parameters using the population pharmacokinetic model, incorporating allometrically scaled parameters and a limited number of plasma concentrations (Guan et al., 2019; Li et al., 2022).

### 2.5 DNA isolation and genotyping

Genomic DNA was extracted from peripheral blood cells by the phenol–chloroform extraction method, as described by Blin and Stafford (1976). The quality of the extracted DNA was assessed by measuring the absorbance ratio at A_260_/A_280_, with values ranging from 1.8 to 2.0 being considered acceptable. The concentrations of extracted DNA were detected using a NanoDrop 2000 Spectrophotometer (Thermo Fisher Scientific, Rockford, IL, United States).

The primary outcomes of interest were SNPs in the genomic DNA, which were measured using DNAs extracted from blood cells in the Agena Sequenom MassARRAY Analyzer 4 system (MALDI-TOF platform). In brief, the regions of the genome containing each SNP in extracted DNA samples (20 ng·mL-1) were amplified by a PCR amplifier (Mastercycler^®^ nexus, Eppendorf, Hamburg, Germany), and then, an extension PCR reaction was performed in a 384-well plate (Thermo Fisher Scientific, Rockford, IL, United States). After that, a single terminator nucleotide base extends the DNA fragment. The terminator bases were desalted and dispensed into a SpectroCHIP^®^ Array (Sequenom, San Diego, CA, United States). The detection of alleles was conducted using a MassARRAY mass spectrometer (Sequenom, San Diego, CA, United States). MassArray Typer 4.0 software (Sequenom, San Diego, CA, United States) was used to analyze the data. Inspection of the clusters was performed to ensure a clear cluster separation with a satisfactory signal-to-noise cut-off. SpectroChip data with a more than 10% call rate in the blank check or with a more than 25% call rate in the blank control were considered invalid and were repeated.

All SNPs associated with dexmedetomidine in this study were selected based on one or more of the following criteria: (1) minor allele frequency (MAF) greater than 0.05 for the Chinese population in the GRCH37 database (http://grch37.ensembl.org/index.html), (2) previously reported associations with DEX, and (3) previously reported functional effects. The candidate SNPs were genotyped using the Agena Sequenom MassARRAY platform (San Diego, CA, United States). The primers of each SNP are presented in Supplementary Table S2.

### 2.6 Statistical analysis

Continuous variables were presented as medians with interquartile range (IQR), while categorical variables were expressed as frequencies (%). The Hardy–Weinberg equilibrium for candidate SNPs was assessed using a χ^2^ test, with a P-value < 0.05 indicating a deviation from equilibrium.

The cumulative incidence of hemodynamic instability was evaluated using the Kaplan–Meier method. Univariate Cox models were fit for those variables for the outcomes, and hazard ratios (HRs), 95% CI, and P values were computed for each variable using the cox.zph function of the survival package in R. Time at zero was recognized as the initiation of DEX administration, and the last follow-up time was defined as the completion of the operation.

Least absolute shrinkage and selection operator (LASSO) regression with 100-repeated fivefold cross-validation was applied to identify the most predictive factors for DEX-associated hemodynamic instability using the R package glmnet. A total of 270 patients were randomly split into the training (60%) and test cohorts (40%). Initially, the lambda sequence was computed, followed by model fitting for each fold omission. The optimal lambda value was determined based on the standard deviation and average error across folds. The elasticnet mixed parameters (0≤α ≤ 1) were used, where α = 1 for the LASSO penalty and α = 0 for the Ridge penalty. After 10,000 iterations of the LASSO model, the frequency of variables was calculated. Variables were ranked based on the frequency, and these were progressively added to the test cohort. The process continued until the area under the receiver operating characteristic curve (AuROC) in the test cohort showed no further significant improvement.

A score-based nomogram model was developed based on multivariate Cox regression analysis. Nomogram is a statistical model that provides individualized risk assessment. The calibration and discrimination performances of the nomogram model were evaluated alongside accuracy, specificity, and sensitivity using the receiver operating characteristic (ROC) curve. The optimal threshold prediction value of the multivariate regression model was determined using the maximum Youden’s index.

Statistical analyses and data management were conducted using R version 4.2.2 (R Foundation for Statistical Computing, Vienna, Austria). Pharmacokinetic estimation was conducted using Phoenix NLME (Version 7.0, Certara L.P. Pharsight), and the non-compartment model was calculated in the WinNonlin mode. Graphs were plotted using GraphPad Prism 8 (GraphPad Software Incorporated, San Diego, CA, United States).

## 3 Results

### 3.1 Pediatric participants’ characteristics

**FIGURE 1:**
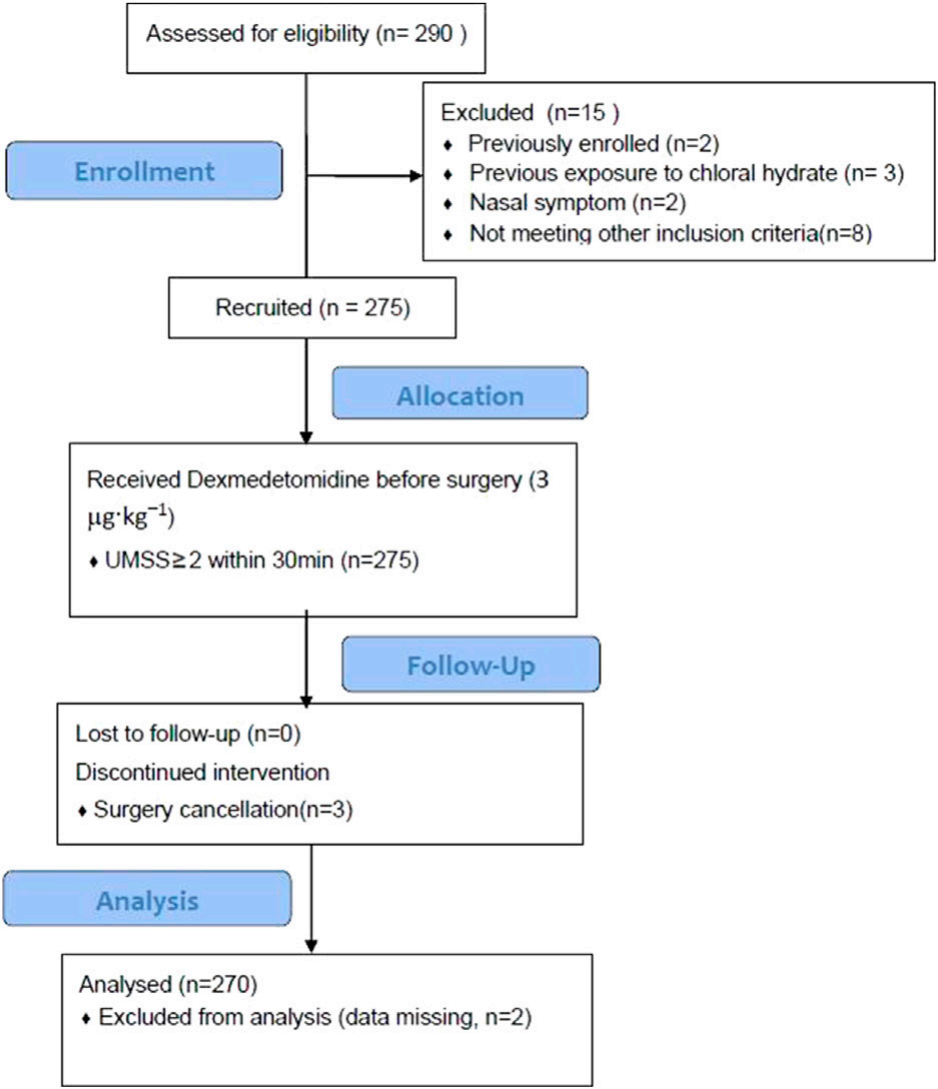
A flow diagram.

In total, 285 potentially eligible pediatric patients received DEX pre-operatively during the study timeframe, and 270 patients met the inclusion criteria. There were 15 dropouts because of previous exposure to DEX (n = 2) or chloral hydrate (n = 3), previous enrollment (n = 2), and nasal symptom (n = 2). The patients’ flow diagram is shown in Figure 1. Data from 270 pediatric participants (median (IQR) aged 22 (8–42) months (146 female and 124 male individuals) are presented in Table 1.

**TABLE 1:** Characteristics of pediatric patients. All values are expressed as N (%) or median (interquartile ranges, IQR).

Characteristic	Total cohort (n = 270)
Age (months)	22 (8–42)
Male/female	124/146
Weight (kg)	11 (8–15)
Height (cm)	83 (71–100)
Disease types	
Hemangioma	158 (58.52%)
CHD	112 (41.48%)
Surgical interventions	
Atrial septal defect (ASD) repair	24 (8.89%)
Ventricular septal defect (VSD) repair	32 (11.85%)
Patent ductus arteriosus (PDA) repair	45 (16.67%)
Pulmonary stenosis	7 (2.59%)
Sclerotherapy	78 (28.89%)
Embolization	30 (11.11%)
Cryotherapy	14 (5.19%)
Others	40 (14.81%)
Concomitant sedatives	
Sufentanil	267 (98.89%)
Propofol	270 (100.00%)
Sevoflurane	270 (100.00%)
Cisatracurium	270 (100.00%)
Albumin (g·L^−1^)	45.10 (43.10–47.45)
Hematoidin (Umol·L^−1^)	3.80 (2.70–5.75)
Creatinine (μmol·L^−1^)	22.00 (17.00–27.00)
Urea (mmol·L^−1^)	3.90 (2.90–4.70)
ALT (U·L^−1^)	17.00 (13.00–23.00)
AST (U·L^−1^)	33.00 (28.00–40.00)
Hemodynamic parameters (baseline)^a^	
Systolic blood pressure (mmHg)	93.00 (88.00–101.00)
Diastolic blood pressure (mmHg)	55.00 (48.25–61.75)
Mean arterial blood pressure (mmHg)	67.67 (62.67–74.00)
Heart rate (bpm)	111.50 (104.00–120.00)
SOP_2_	100 (100–100)
Sedation onset time (min)	16 (12–22)
Recovery time (min)	55 (40–70)
Discharge time (min)	115 (100–140)

CHD, congenital heart disease; ALT, alanine aminotransferase; AST, aspartate amino transferase; SOP_2_, oxyhemoglobin saturation.

^a^ The data collected before dexmedetomidine administration.

### 3.2 Hemodynamic profiles

**FIGURE 2:**
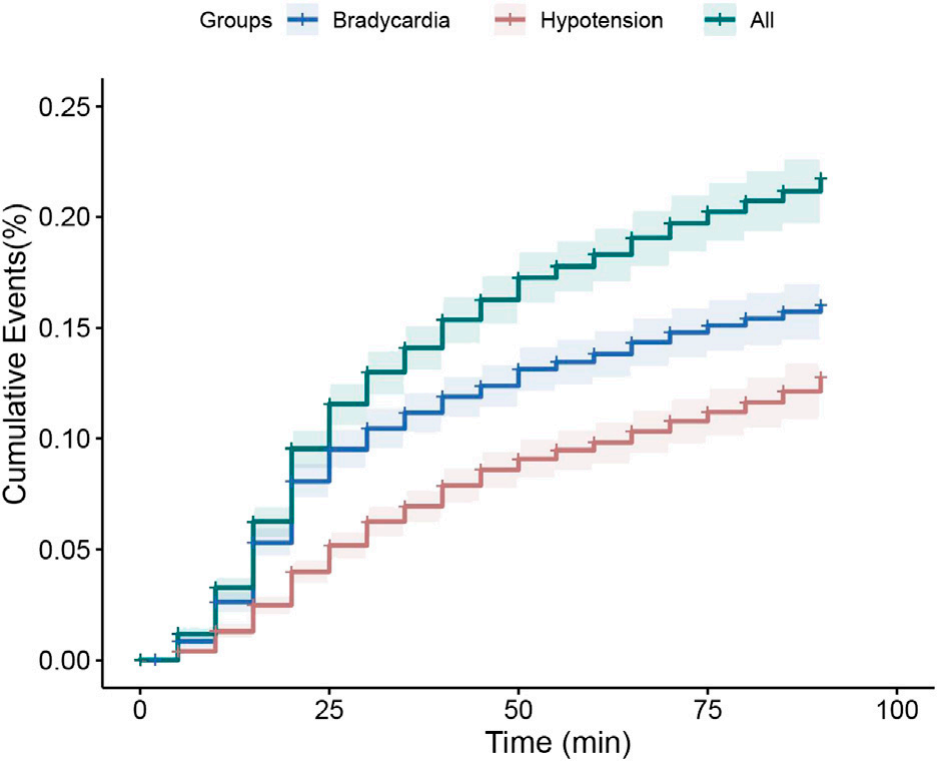
Kaplan–Meier curve for estimate of dexmedetomidine associated hemodynamic instability cumulative incidence of dexmedetomidine therapy within 90 min. Hemodynamic instability was the composite of hypotension (20% reduction in systolic/diastolic blood pressure, red lines), and/or bradycardia (20% reduction in heart rate, blue lines), and all the patients with hemodynamic instability (green lines). The solid line represents cumulative incidence, and the dash area represents the 95% confidence interval.

In this study, hypotension and bradycardia were commonly observed but were not life-threatening side effects, and only four patients required interventions to treat hypotension/bradycardia. One patient exhibited oxygen saturation below 93% at 45 min post-induction of anesthesia. Using the Kaplan–Meier analysis, the cumulative incidence of hemodynamic instability within 90 min after DEX administration was estimated to be 16.3% at 90 min (Figure 2). Among the enrolled patients, at least one episode of hemodynamic instability occurred in 52 patients (209 events). After achieving the target UMSS at sedation onset time [median (IQR): 16 min (12–22 min)] after intranasal DEX administration, they experienced induction anesthesia and received fixed doses of propofol (2 mg·kg^−1^), sufentanil (0.3 mcg·kg^−1^), and cisatracurium besylate (0.2 mg·kg^−1^). The first hemodynamic instability commonly occurred at the median time of 20 min (15–30 min) after DEX administration, and approximately 14.4% (39 out of 270) of children experienced it after the initiation of anesthesia induction.

### 3.3 Pharmacokinetic profiles

Plasma samples from 270 pediatric patients were collected at 60 min after DEX administration. The median concentration level at 60 min after DEX initiation was 0.429 ng·mL^−1^ (IQR: 0.332–0.523 ng·mL^−1^). Median plasma concentrations at different time points (0–6 h) along with fitted PK profiles were simulated using a validated population pharmacokinetic model (shown in Supplementary Figure S1). All the model-derived pharmacokinetic parameters are shown in Table 2.

**TABLE 2:** Pharmacokinetic characteristics of pediatric patients. All values are expressed as N (%) or median (interquartile range).

Characteristic	Total cohort (n = 270)
Dexmedetomidine concentrations	0.429 (0.332–0.523)
Pharmacokinetic profiles	
Absorption rate (Ka)	0.38 (0.24–0.56)
Half-life time (t_1/2_, h)	1.84 (1.36–2.83)
t_max_ (min)	54.00 (36.00–75.00)
C_max_ (μg·L^−1^)	0.79 (0.65–1.04)
Clearance (L·h^−1^)	0.94 (0.64–1.37)
Patients with hypotension^a^	0.76 (0.51–1.27)
Patients without hypotension^a^	0.98 (0.80–1.51)
Patients with bradycardia^a^	0.74 (0.56–1.30)
Patients without bradycardia^a^	0.95 (0.72–1.66)
Volume of distribution (L)	2.67 (2.08–3.34)
AUC_0–0.5_ (μg·h·L^−1^)	0.23 (0.16–0.32)
AUC_0–1_ (μg·h·L^−1^)	0.60 (0.45–0.82)
AUC_0–2_ (μg·h·L^−1^)	1.27 (1.00–1.65)
AUC_inf_ (μg·h·L^−1^)	3.19 (2.18–4.65)

^a^ The data evaluated after dexmedetomidine administration within 35 min.

AUC_0–0.5_, area under the concentration–time curve from 0 h to 0.5 h.

AUC_0–1_, area under the concentration–time curve from 0 h to 1 h.

AUC_0–2_, area under the concentration–time curve from 0 h to 2 h; AUC_inf_, area under the concentration–time curve from 0 h to infinity.

Subgroup analysis on patients with hypotension or bradycardia comparing patients without events showed a significant difference in DEX clearance after 35-min DEX initiation [hypotension vs. normotension: 0.76 vs. 0.98 (*P* = 0.029); bradycardia vs. normal: 0.74 vs. 0.95 (*P* = 0.0078)] (Table 2).

### 3.4 Univariate analysis

**FIGURE 3:**
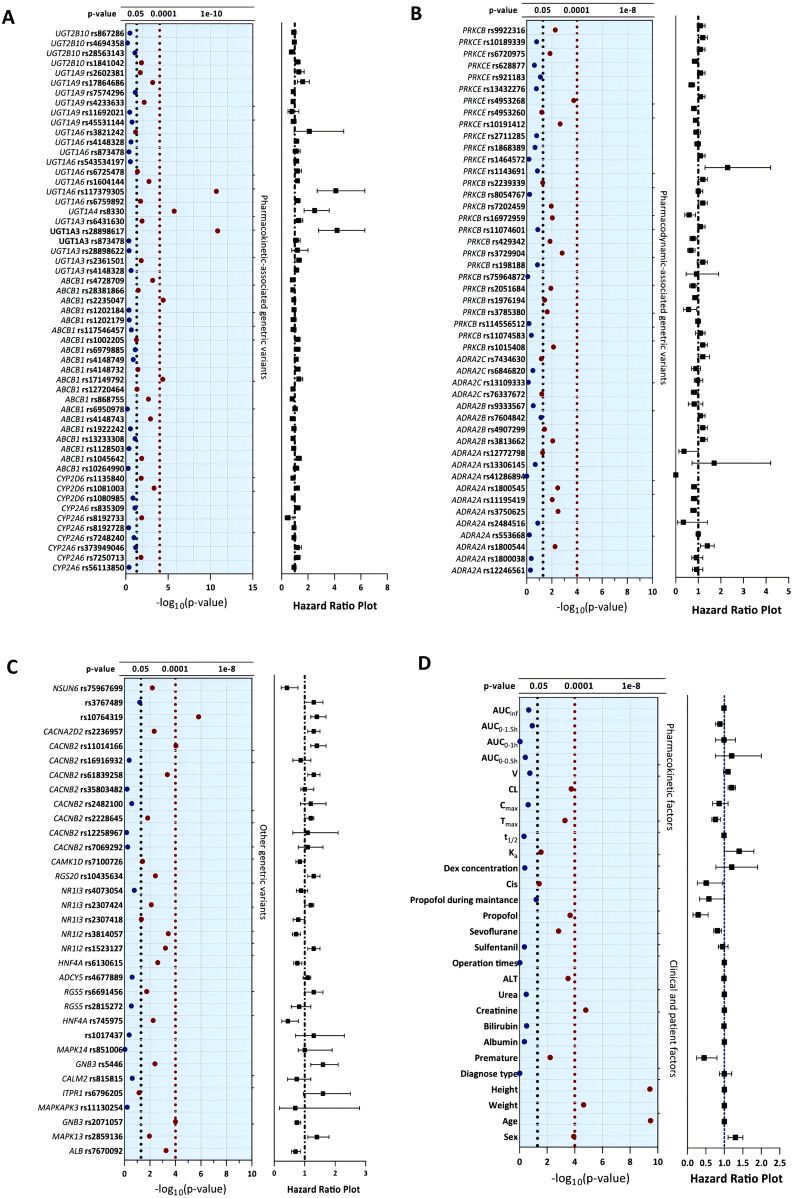
Univariate analysis of clinical, pharmacokinetic and pharmacogenetic variables with dexmedetomidine-induced hemodynamic instability. The blue solid dots represent the features with *P* values higher than 0.05, and the red solid dots represent the significant features with *P* values lower than 0.05.

All allelic distributions for the candidate SNPs conformed to the Hardy–Weinberg equilibrium (*p* > 0.05). Among the candidate SNPs, univariate analysis showed that 21 of the remaining 134 tag SNPs could serve as significant predictors for DEX-induced hemodynamic instability (FDR <0.05) and were used for the next multivariable Cox analysis. Of the clinical variables tested, sex (*p* < 0.001), age (*p* < 0.001), weight (*p* < 0.001), height (*p* < 0.001), premature birth (*p* = 0.006), creatinine (*p* < 0.001), ALT (*p* < 0.001), cisatracurium besylate (*p* = 0.04), and sevoflurane (*p* = 0.002) had significant correlations with DEX-induced hemodynamic instability. All the concomitant medications were recorded and analyzed in the univariate analysis, and only concomitant propofol (*p* < 0.001) had a significant impact on DEX-associated hemodynamic instability. In the further subgroup analysis, patients with concomitant sevoflurane were divided into three dosage groups (<1.0, 1.0–2.0, and >2.0%), and the risk of hemodynamic instability increased with the dosage of sevoflurane (*p* = 0.004). Of the pharmacokinetic variables tested, peak time (*p* < 0.001) and DEX clearance (*p* < 0.001) demonstrated significant associations with outcomes. The results of univariate analysis are shown in Figure 3 and Table 3.

**TABLE 3:** Genetic variants associated with dexmedetomidine-induced hemodynamic instability in this retrospective study.

Chr	Gene	SNP	Major/minor allele	MAF	SNP is	Genetic model	β	*p*-value
4	UGT2B10	rs1841042	A/G	0.19	Intron variant	AA vs. AG + GG	0.15	0.017
19	CYP2A6	rs8192733	G/C	0.48	3 prime UTR variant	GG vs. GC + CC	−0.73	0.013
2	ADRA2B	rs3813662	A/C	0.14	3 prime UTR variant	AC vs. AA + CC	0.21	0.0086
3	CACNA2D2	rs2236957	A/G	0.49	Intron variant	AA vs. GG + GA	0.23	0.0046
3	NR1I2	rs3814057	C/A	0.49	3 prime UTR variant	CC vs. AA + AC	−0.33	0.00036
10	CACNB2	rs10764319	C/T	0.49	Intron variant	CT vs. CC + TT	0.36	1.50E-06

Chr, chromosome; MAF, minor allele frequency in the study sample.

### 3.5 LASSO regression and multivariate analysis

**FIGURE 4:**
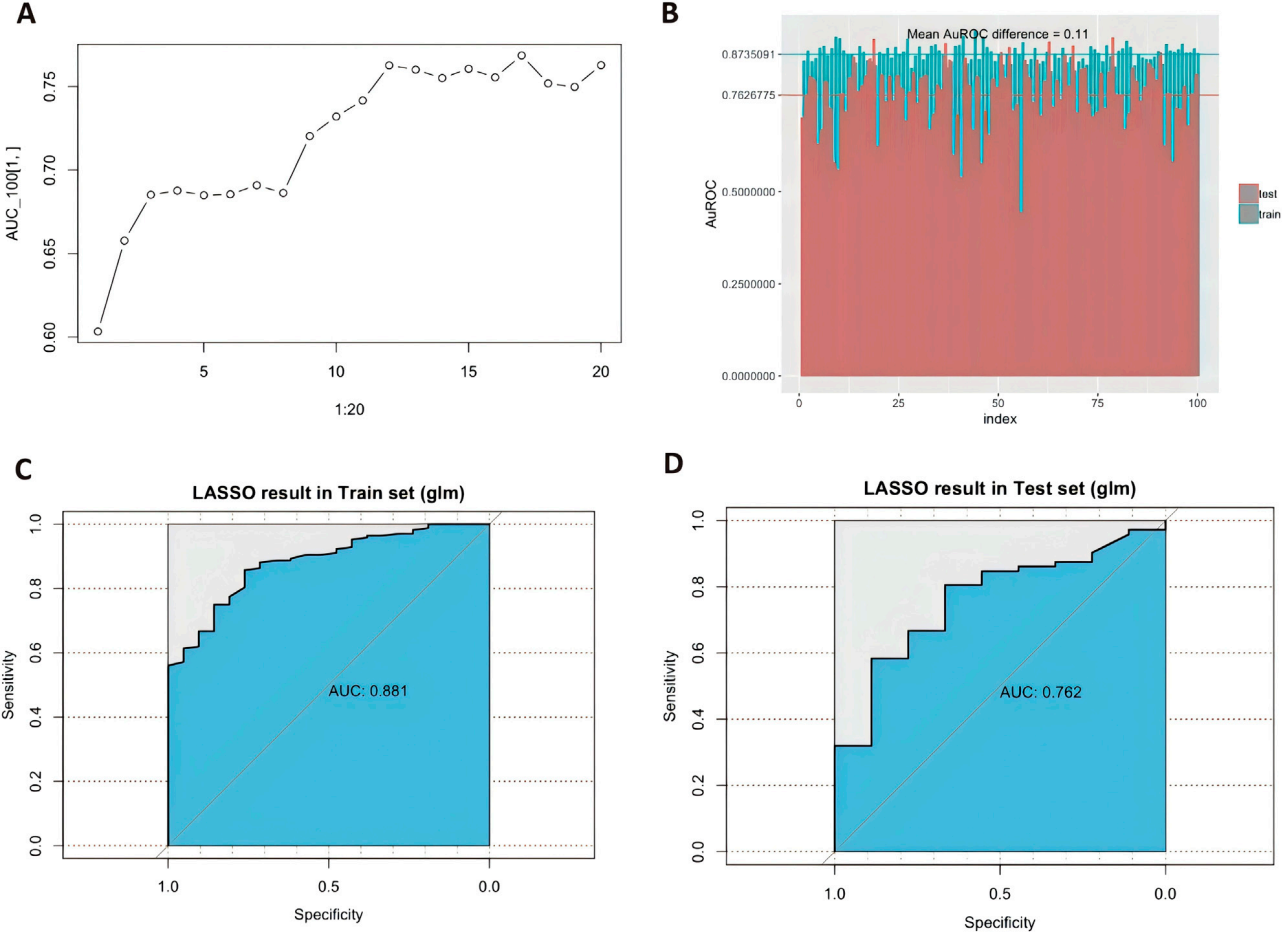
LASSO regression screening and the model diagnostic plots. **(A)**.Area under the receiver operating characteristic curve (AuROC) in the test cohort showed no further significant improvement after adding the significant factors step by step. **(B)**. Different area under the curve (AUC) values across fivefold cross-validation (n = 100 times). The graph’s horizontal axis shows the dataset number, and the vertical axis shows the AuROC value. The blue columns represent the training datasets, and the red ones are test datasets. Mean AuROC on the training cohort and validation cohort are 0.873 ± 0.042 and 0.762 ± 0.063, respectively. The mean difference between training datasets and testing datasets is 0.11. Receiver operating characteristic curve for predicting dexmedetomidine treatment outcome in the training cohort [**(C)**, n = 162] and test cohort [**(D)**, n = 108] with the predictors.

The complete dataset was randomly split into training and validation datasets. The training dataset comprised 60% of the total patients, and the remainder constituted the test cohort. LASSO regression was used to evaluate the frequencies of variables from all the candidate factors. The changes in areas under the curve (AUCs) after adding the variables and the average AuROC values for the training and test datasets over 100 replications are shown in Figure 4.

**FIGURE 5:**
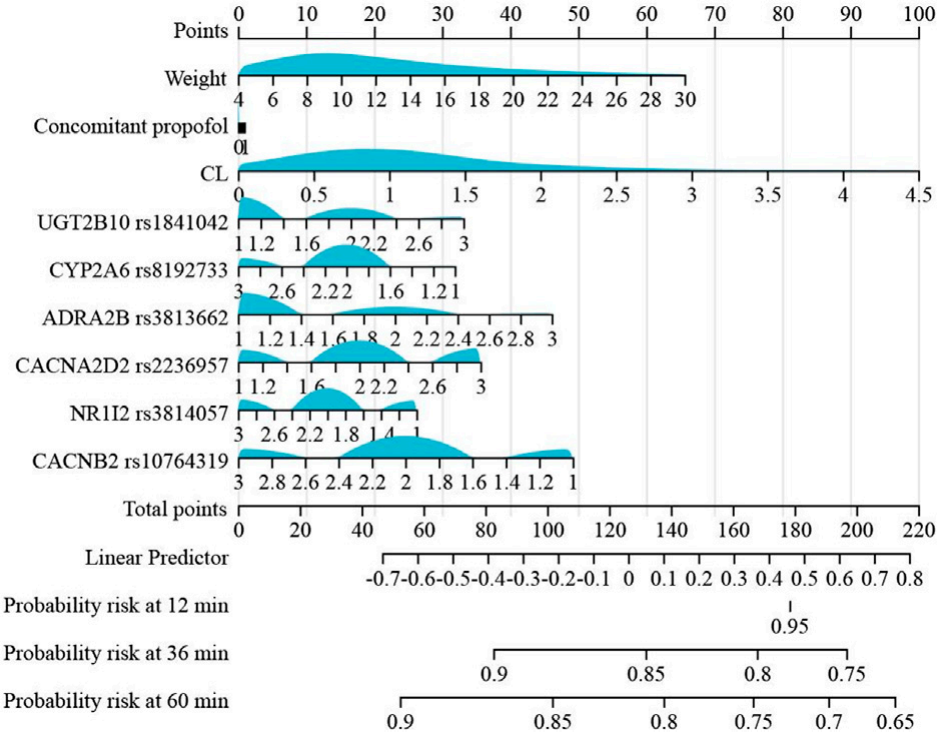
Developed predictive nomogram.

Multivariable Cox models were analyzed, and the following variables were retained in the model: weight, dexmedetomidine clearance, concomitant propofol at induction anesthesia, *UGT2B10* rs1841042, *CYP2A6* rs8192733, *ADRA2B* rs3813662, *CACNA2D2* rs2236957, *NR1I2* rs3814057, and *CACNB2* rs10764319. In the training cohort, the AUC values for the training and the test cohorts were 0.881 and 0.762, respectively (Figures 4C, D). Figure 5 shows the nomogram based on the above predictors containing 270 pediatric patients. The calibration curves demonstrated an optimal consistency between the nomogram-predicted and the actual observed risk probability (Supplementary Figure S2).

## 4 Discussion

Episodes of DEX-induced hemodynamic instability are seen in 25%–47.6% of pediatric patients (Okello et al., 2018). There is significant inter-individual variability in the hemodynamic response to the administration of DEX (Adefurin et al., 2015). The mechanism underlying this instability has remained incompletely understood. This study aims to investigate the clinical, pharmacokinetic, and pharmacogenetic variables that influence hemodynamic instability during DEX pre-operative anesthesia. Among the clinical and pharmacokinetic characteristics, weight, DEX clearance, and concomitant propofol administration were identified as independent factors influencing DEX-related hemodynamic instability. Understanding DEX pharmacokinetics is essential for a comprehensive insight into the mechanisms of hemodynamic instability. In this context, DEX clearance was retained in the multivariable Cox model.

Previous evidence has established pharmacokinetic models as predictive of dexmedetomidine clearance, including the use of allometrically scaled and maturity models (weight and postmenstrual age) (Li et al., 2022). Patient weight is directly correlated with downstream effects in the elimination phase and used to simulate the plasma concentrations for individual children (Li et al., 2022); this is why the patient weight can also be retained as a predictor in the final model. Subsequently, subgroup analysis for DEX clearance was conducted on patients with hypotension or bradycardia compared with patients without episodes after 35-min DEX initiation [hypotension vs. normotension: 0.76 vs. 0.98 (*p* = 0.029); bradycardia vs. normal: 0.74 vs. 0.95 (*p* = 0.0078)]. Commonly, patients received induction anesthesia (concomitant propofol) after achieving the target UMSS at onset time. Findings from meta-studies have shown that propofol might increase the risk of hypotension and DEX might increase the risk of bradycardia in patients (Sneyd et al., 2022). These findings suggest that DEX plasma levels may decline significantly after the peak concentration time [median (IQR): 54 min (36–75 min)] due to accelerated elimination kinetics. Consequently, delayed hemodynamic instability (occurring 35 min after DEX initiation) is likely to occur in patients with lower DEX clearance (resulting in higher plasma concentration levels), following propofol induction. In hemodynamic instability, it appeared that hypotension/bradycardia occurred at least 35-min after DEX administration, coinciding with the start of propofol induction. Regarding the peak concentration and peak time herein, slight differences were found, while a study showed a median peak time for 37 min (30–45 min) and a peak concentration of 0.54 ng·mL^−1^ (Simpao et al., 2020). A possible cause of this is that plasma DEX concentration vs. time profiles are simulated by only single observations, which may influence pharmacokinetic estimates. Another possible cause is that hypotension could have reduced clearance and altered the plasma concentration in the second blood samples taken after the induction of anesthesia (Simpao et al., 2020). Additionally, hypotension occurred commonly in patients with a median age of 23 months (range 2–35 months) in the current study. Prolonged fasting time causes volume deletion and increased the incidence of low blood pressure, especially in younger anesthetized children. Therefore, it is important to carefully consider preoperative fasting times in this population, especially in younger children (Dennhardt et al., 2016).

The impact of a_2_-adrenoceptors’ gene polymorphisms, such as *ADRA2A, ADRA2B*, and *ADRA2C*, on the vascular response, have been widely studied in patients (Murakami et al., 2000; Kaur and Singh, 2011; Lu et al., 2015). Findings from several studies have shown that *ADRA2A C-1291G, ADRA2B 301–303 I/D*, and *ADRA2C del322-325* (Murakami et al., 2000; Lu et al., 2015) have no significant association with blood pressure or hypotension. Concordant with this observation, candidate SNPs in *ADRA2A* and *ADRA2C*, in our study, do not alter sensitivity to vascular response caused by dexmedetomidine. With regard to *ADRA2B*, we first found that the carriers of *ADRA2B* rs3813662 CC + AA genotypes can increase the risk of hemodynamic instability even more significantly than AC genotypes. This SNP is located in 3′-untranslated regions (3′-UTRs), which are best known to regulate various fates of mRNAs (Mayr, 2017).

*CACNB2* and *CACNA2D2* encode the intracellular beta-2 and alpha-2/delta-2 subunits of a calcium channel (Scholl et al., 2013; Simonyte et al., 2018), and previous studies reported that mutations in *CACNB2* and *CACNA2D2* can influence intracellular calcium homeostasis and alter blood pressure (Murakami et al., 2000; Moosmang et al., 2003). Studies have shown that CACNB2 was associated with DBP, systolic pressure, mean arterial pressure, and hypertension (Levy et al., 2009; Hong et al., 2013). In our study, *CACNB2* rs10764319 (*p* < 0.001) and *CACNA2D2* rs2236957 (*p* = 0.0046) showed significant differences in DEX-induced hemodynamic instability. Both of them are located in the non-coding region, in which the intronic variation can enhance transcription, encode RNA genes, and allow alternative splicing.

Dexmedetomidine undergoes extensive metabolism in the liver, particularly involving CYP2A6 (human cytochrome P450 2A6) and UGT2B10 (UDP glucuronosyltransferase family 2 members B10). NR1I2 (human pregnane X receptor) is a nuclear receptor that is classified as a xenoreceptor. In our study, DEX-induced hemodynamic instability was found to be associated with *CYP2A6, UGT2B10*, and *NR1I2* genes, given the function in DEX disposition. We speculated that the mutations of *NR1I2* could regulate the transcription of metabolized enzymes, and CYP2A6 and UGT2B10 could influence the metabolism and disposition of DEX, alter the concentration of DEX, and subsequently lead/avoid toxic variability in patients (Kaivosaari et al., 2008; Kohli et al., 2012; Wang et al., 2018). However, genetic variations in CYP2A6 in previous studies, namely, CYP2A6*4; CYP2A6*1/*1; CYP2A6*1/*4; CYP2A6*4/*451; and *CYP2A6* alleles *2, *4, *9, *12, and *17 (Wang et al., 2018) were found to have no significant influence on the pharmacokinetics of DEX in previous studies.

Our study has several limitations that we must acknowledge. The current study is an observational study and was performed in a single center. The results require verification in larger, prospective studies. It is to be noted that we recruited pediatric patients with hemangioma (n = 158) or congenital heart disease (n = 112) in this study. These patients have normal liver function. The precise type of pathology for congenital heart disease or its state of compensation is not known. Consequently, the analysis of any cardiovascular repercussions linked to the use of DEX has a very limited value. Although the types of disease did not show significant differences in correlation analysis, they still need to be taken into account. Another limitation to this study is the reliance on adult reference data for the comparison of candidate single-nucleotide polymorphisms (SNPs). This approach may not fully account for the phenotypic changes occurring in children as they still undergo developmental processes, including alterations in gene expression patterns and organ growth. These age-related physiological changes can lead to differences in gene expression between children and adults, which may influence the interpretation of genetic data. Moreover, we did not analyze the correlation between the actual DEX concentration and hemodynamic outcomes because the plasma samples were collected at 60 min post-administration but not at the onset time. Finally, further investigations related to the SNP interactions of reported genes and functional analysis in a larger sample size need to be performed.

This study has several strengths. This current study comprehensively determined the pivotal genetic polymorphisms of drug targets such as a2-ARs, PKC, and calcium channels, all of which might influence the side effects of DEX-induced hemodynamic instability. Second, to avoid confounding, clinical factors, sex, weight, age (months), types of disease, and statistical significance for each SNP have been reanalyzed by the multivariate Cox model. Third, the association of DEX pharmacokinetics simulated by a validated population pharmacokinetic model with hemodynamic instability was also taken into account.

## 5 Conclusion

This study demonstrated that weight, DEX clearance, concomitant propofol, and the SNPs in *ADRA2B, UGT2B10, CYP2A6, CACNA2D2, NR1I2*, and *CACNB2* were related to DEX-induced hemodynamic instability. After the intranasal administration of DEX, a low DEX clearance during propofol induction was associated with hemodynamic instability. The nomogram performed well in the internal validation. Clinicians could perform an individualized risk prediction of DEX-associated hemodynamic instability with this easy-to-use nomogram and prepare preventive interventions that could improve the safety and surgical outcomes in pediatric patients.

## Data Availability

The data presented in the study are deposited in the figshare repository, accession number: https://doi.org/10.6084/m9.figshare.28006601.v2.

## Supplementary material

SUPPLEMENTARY FIGURE S1 Median plasma concentrations at different time points (0–6 h) along with fitted PK profiles were simulated using a validated population pharmacokinetic model.

SUPPLEMENTARY FIGURE S2 Calibration plots of hemodynamic instability based on Cox regression modeling in the whole dataset.
