# Supplementary material for: Pharmacogenetic and pharmacokinetic factors for dexmedetomidine-associated hemodynamic instability in pediatric patients
Source: Front Pharmacol. 2025 Jan 7;15:1515523. doi: 10.3389/fphar.2024.1515523 (PMC11745869; doi:10.3389/fphar.2024.1515523)
Supplement: Supplementary file 1 [file Table1.docx]

| 0 | Awake/alert |
| --- | --- |
| 1 | Minimally sedated: tired/sleepy, appropriately responds to verbal conversation and/or sounds. |
| 2 | Moderately sedated: somnolent/sleeping, easily aroused with light tactile stimulation |
| 3 | Deeply sedated: deep sleep, arousable only with significant physical stimulation |
| 4 | Unarousable |

**Supplemental Table 1. University of Michigan Sedation Scale (UMSS)**
