# Supplementary figures and images for: Pharmacogenetic and pharmacokinetic factors for dexmedetomidine-associated hemodynamic instability in pediatric patients
Source: Front Pharmacol. 2025 Jan 7;15:1515523. doi: 10.3389/fphar.2024.1515523 (PMC11745869; doi:10.3389/fphar.2024.1515523)

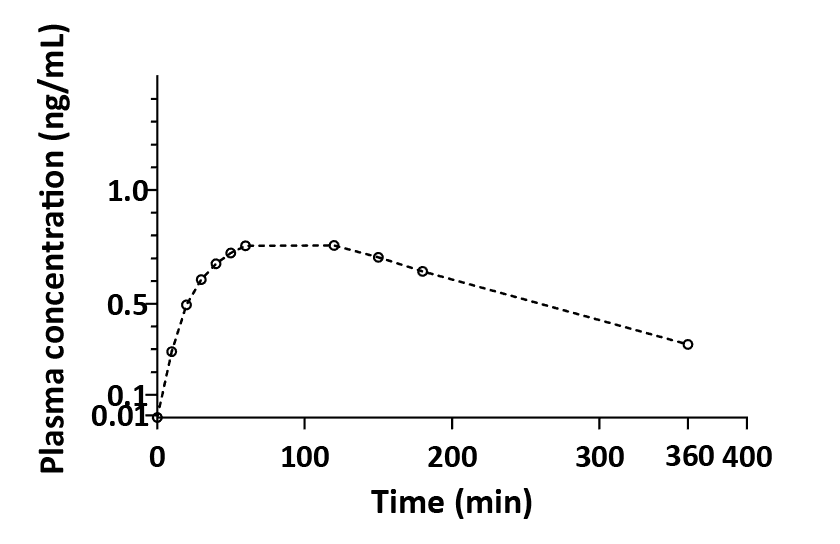

Supplement: Supplementary file 2 [file Image1.jpeg]

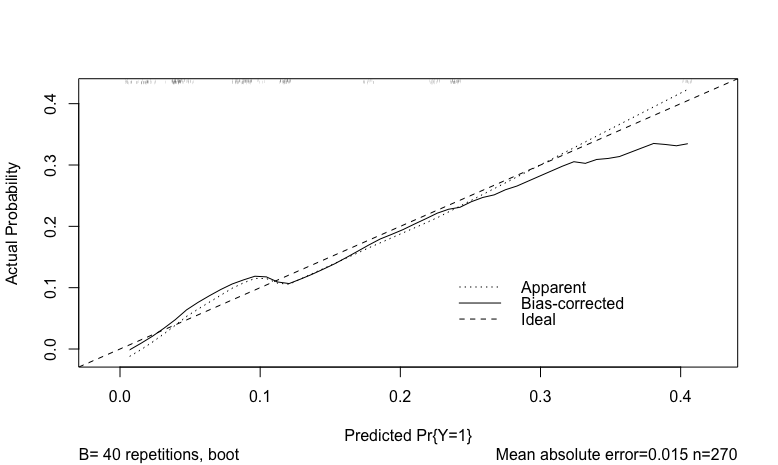

Supplement: Supplementary file 4 [file Image2.tiff]
