# Supplementary material for: Pharmacogenetic and pharmacokinetic factors for dexmedetomidine-associated hemodynamic instability in pediatric patients
Source: Front Pharmacol. 2025 Jan 7;15:1515523. doi: 10.3389/fphar.2024.1515523 (PMC11745869; doi:10.3389/fphar.2024.1515523)
Supplement: Supplementary file 3 [file Table2.docx]

**Supplemental Table 2. The primers of candidate SNPs**

| **Gene** | **SNP** | **Forward primer sequence** | **Reverse primer sequence** | **Extension primer sequence** |
| --- | --- | --- | --- | --- |
| CYP2A6 | rs56113850 | ACGTTGGATGAGTTAAAGGCATCTGTCTCG | ACGTTGGATGCTGGTACCTAACCTTCCTGA | CGGTGGCCGGATAGGAACT |
| CYP2A6 | rs373949046 | ACGTTGGATGTGAAGCCCACTCTCAGTGC | ACGTTGGATGGCCATAATATTCCACCCTTC | AGCTGGAGGTCGGTA |
| CYP2A6 | rs7250713 | ACGTTGGATGCCAGACCCTCTCTGTTTCTA | GGAGGAAATAAAAATGGTGAAA | ACGTTGGATGGAGATCTGGGAGGAGGAAAT |
| CYP2A6 | rs7248240 | ACGTTGGATGATCCTGCCCCCAGTCTTAG | ACGTTGGATGTGTGCCGGTGCAGGTCTGGT | GGCCCTCTCCCAAGCCC |
| CYP2A6 | rs8192728 | ACGTTGGATGATGATATGGCTCCGCCTATG | ACGTTGGATGACCTGGTTAACAGGATCCTA | ccatAACAGACATCAGCCATTCGCATT |
| CYP2A6 | rs8192733 | GGAAGAGAAGAAACAGAAG | ACGTTGGATGCTCGGAAGCACCTTATCAAG | ACGTTGGATGCAGGATGGCGGAAAGGAAG |
| CYP2A6 | rs835309 | ACGTTGGATGCTACAACTATCTATATAACTG | ACGTTGGATGGAAGACAACCTCCTGGTAAC | ACAACTGAGATTTAGAAGGAAAA |
| CYP2D6 | rs1080985 | ACGTTGGATGGCACCCAATCCCAGCTAATT | ACGTTGGATGCTTCACCCCAGGAATTCAAG | ccgAATTTTGTATTTTTTGTAGAGACC |
| CYP2D6 | rs1081003 | ACGTTGGATGTCCCACGGAAATCTGTCTCT | ACGTTGGATGTGCCCATCACCCAGATCCT | ACCCAGATCCTGGGTTT |
| CYP2D6 | rs1135840 | ACGTTGGATGACTAGGTACCCCATTCTAGC | ACGTTGGATGCCATGGTGTCTTTGCTTTCC | tgtaTTTGCTTTCCTGGTGA |
| UGT1A3 | rs2361501 | ACGTTGGATGTCTCAGGTCTGTATTGGTGC | ACGTTGGATGGATAAGTAAATATGGAAGC | aaacTGTTCCAGGCAAAACACTTTTT |
| UGT1A3 | rs873478 | ACGTTGGATGGTCACGTGACACAGTCAAAC | ACGTTGGATGAGGTTCGCCCTCTCCTACTT | AACATTAACTTGGTGTATCGATT |
| UGT1A3 | rs28898622 | ACGTTGGATGTGTTGCCCTTCCAGTTTTTC | ACGTTGGATGACCTATGTATCAAACCTGC | tctgATACTTTAAGTTCTAGGGTACAT |
| UGT1A3 | rs28898617 | ACGTTGGATGAGTCAGCTGTCCGTGTCTTC | ACGTTGGATGCTGGACACTGAGGAGAAGCA | cctgcGATGGCCACAGGACTCC |
| UGT1A3 | rs6431630 | ACGTTGGATGTGTTGGTGGTGCCCATTGAT | ACGTTGGATGTGGTGGCCTCTGGCATGGA | TCAGCATGCGGGAGG |
| UGT1A4 | rs8330 | ACGTTGGATGACCAATAATGGTCAGTCCTC | ACGTTGGATGCTAAAGGTACAAAGCTTCCC | CAGTCCTCATCTCTGTC |
| UGT1A6 | rs117379305 | ACGTTGGATGGATGGACTAGGACTGATGTG | ACGTTGGATGCTTCCCAATGAAGGACATGC | GACTCATGGAGACTGTT |
| UGT1A6 | rs6759892 | ACGTTGGATGTTAACTCTTTCCAGGATGGC | ACGTTGGATGTGCCCCAAAGTGCTAAGAAG | ttGCCTGCCTCCTTCGC |
| UGT1A6 | rs1604144 | ACGTTGGATGAATAGTTTGAGTGGAACTGG | ACGTTGGATGCTCTCATCAAAGAAAAGCCC | AATGGTTGGTAGAATTCAG |
| UGT1A6 | rs543534197 | ACGTTGGATGTGGAGTGCAATGGCACGATG | ACGTTGGATGTGTAGTCCTAGCTACTTGTG | gctaACTGCAACCTCTGCCTC |
| UGT1A6 | rs6725478 | ACGTTGGATGCCCCATTAGATTTAAAACTCC | ACGTTGGATGTCTGTGCAGGAAACTTATGG | gaAGATGTCTATTATGGCAACTTTTTAT |
| UGT1A6 | rs3821242 | ACGTTGGATGAGTCAGCTGTCCGTGTCTTC | ACGTTGGATGCTGGACACTGAGGAGAAGCA | ggAGTCCTGTGGCCAGCC |
| UGT1A6 | rs873478 | ACGTTGGATGGTCACGTGACACAGTCAAAC | ACGTTGGATGAGGTTCGCCCTCTCCTACTT | gACATTAACTTGGTGTATCGATT |
| UGT1A6 | rs4148328 | ACGTTGGATGCCCCATTAGATTTAAAACTCC | ACGTTGGATGTCTGTGCAGGAAACTTATGG | CCATTAGATTTAAAACTCCAATTTA |
| UGT1A9 | rs11692021 | ACGTTGGATGTCTCTTAGGGTTCTCAGACG | ACGTTGGATGTGTTCCTCCAAGTGCATGAT | taccTGACTTTCAAGGAGAGAGTA |
| UGT1A9 | rs45531144 | ACGTTGGATGCAGGTCCCAAAGACCCTTAC | ACGTTGGATGTGGCTACTGCCAACTGTTCA | CCCTTACCTCTCTTCTTTA |
| UGT1A9 | rs4233633 | ACGTTGGATGTTCCAAGATACATGGGCCTC | ACGTTGGATGCTCCATGCAGTGGTGTTTTG | gggtGAGTCCTAGACGTGGGA |
| UGT1A9 | rs7574296 | ACGTTGGATGTAACAGACCCCGTTAACCTC | ACGTTGGATGATCACATGGAATGTTCCTC | GGTACTTAGCCAGCAC |
| UGT1A9 | rs17864686 | ACGTTGGATGATTCTCCAAACCCCTGTCAC | ACGTTGGATGATCACGGGTTTGGGATACTC | ACAAAGTCAGTTCGCAA |
| UGT1A9 | rs2602381 | ACGTTGGATGCAAAGTTTTCAAACATACAC | ACGTTGGATGACCTAAGAATGGATAAGTGG | GGATAAGTGGGTGCTC |
| UGT2B10 | rs1841042 | ACGTTGGATGCCTTTTTAGGAACTTAGAG | ACGTTGGATGGCATATTAGAACGTGCTCAAC | AGGATTGCATTTTCACATCTTTCTT |
| UGT2B10 | rs28563143 | ACGTTGGATGAACAAGCAAAGCTAGTGCTG | ACGTTGGATGCACCACTTTTGTCCACCAAC | agAAGCTAGTGCTGCTATTCAA |
| UGT2B10 | rs4694358 | ACGTTGGATGCTGGAAAACCTGATAGATAG | ACGTTGGATGCAGGAAGAAAGAAATCTTGC | GAACACCTGATAGATAGGAATACTTCAG |
| UGT2B10 | rs867286 | ACGTTGGATGCCATATGGACCACAATGCGG | ACGTTGGATGCCAGAAAAATCCTTCCCTCC | CCGACAAATCCTTCCCTCCTGGCAGCCT |
| ABCB1 | rs10264990 | ACGTTGGATGTCACCAGTTTGTAGGACCTC | ACGTTGGATGGAGACTAAGGGAGAAGCATC | tcagTATCCTGGTTCTCCTACTTC |
| ABCB1 | rs1045642 | ACGTTGGATGAAGGCATGTATGTTGGCCTC | ACGTTGGATGGCTGAGAACATTGCCTATGG | tgtttGCCTCCTTTGCTGCCCTCAC |
| ABCB1 | rs1128503 | ACGTTGGATGAGCCACTGTTTCCAACCAGG | ACGTTGGATGTTTCTCACTCGTCCTGGTAG | acctCCCACTCTGCACCTTCAGGTTCAG |
| ABCB1 | rs13233308 | ACGTTGGATGTTTCAATGATCCAGGTGAGC | ACGTTGGATGATCTTGTTGGTGCTACCCTC | tgGGGAATAATGAAAAGTGCTTAGACT |
| ABCB1 | rs1922242 | ACGTTGGATGGTACAATTCTTACATACGCAC | ACGTTGGATGGATAAGGAATAAGGATAGG | ATAAGGATAGGATATATTCCTTTAC |
| ABCB1 | rs4148743 | ACGTTGGATGTTCTCAAAGACCAGATGCCC | ACGTTGGATGTCAGTGTCTTTAATGCCACC | ATGCCCACCCAACTG |
| ABCB1 | rs6950978 | ACGTTGGATGAAGGGCCTGTTTCAGTTGAG | ACGTTGGATGCTTGCCGTTGACTTTTGTCC | CCCCTCTTTTCCATACC |
| ABCB1 | rs868755 | ACGTTGGATGAACCCCCTTAACCAATACAG | ACGTTGGATGTCTGATGCTATAACACTTC | tttaACTTTTTCATACCTTCAAAGATAT |
| ABCB1 | rs12720464 | ACGTTGGATGGCCTATGATCTCTGTTTTCAC | ACGTTGGATGAGGCCATTCAAAAGGATAC | ggaaAAGATTACATAAATGAAAGGTGA |
| ABCB1 | rs17149792 | ACGTTGGATGCAATCTATCCACTCCCTTAC | ACGTTGGATGACCTGGAAGACTACTAAACC | gggaCTGGAAGACTACTAAACCAATTAA |
| ABCB1 | rs4148732 | ACGTTGGATGTAGCCAATACAGCAACAGAC | ACGTTGGATGTGGCTAACTCACTAAGCGAC | tAGTTGTGTGAAATGGTGA |
| ABCB1 | rs4148749 | ACGTTGGATGAGATGACACCACTTGGAGAC | ACGTTGGATGCCTTTAAACAACAGTCCCAC | TGGATAAAGTCTGAGAGC |
| ABCB1 | rs6979885 | ACGTTGGATGGTACATGTACCCCCTAAGTC | ACGTTGGATGGATTATGGATGCTCAACCTG | TATTAAAAGTCCAAAATTAGATTTTTT |
| ABCB1 | rs1002205 | ACGTTGGATGCTCAGGCACTTCTTGGGTTA | ACGTTGGATGGTCTCTCCCATATACAACAG | tgaAGCTTCTAATTGTATCCCAAAG |
| ABCB1 | rs117546457 | ACGTTGGATGCAGTCCACTGGTCACAATAC | ACGTTGGATGTGAAGGAGCATGAATCCCAC | aaagACTCCTATGGGTGGTAC |
| ABCB1 | rs1202179 | ACGTTGGATGAGTGCCCAACCTGTTGTATC | ACGTTGGATGTCTGGCTTGTGTAGTAGCTC | CTATAACTTAGCATATTCAAAAAATTTA |
| ABCB1 | rs1202184 | ACGTTGGATGTTCCTCCCCAATCGAGAAAG | ACGTTGGATGGTGGGAAAGTGAATAGACTAC | GAGAGGAAAGAATATGTATGTTACA |
| ABCB1 | rs2235047 | ACGTTGGATGGGTTGCTAATTTCTCTTCAC | ACGTTGGATGCAAATAAACAGCCTGGGAGC | ccACCAGCCCCTTATAAATCAA |
| ABCB1 | rs28381866 | ACGTTGGATGTCCCAGAGGGTCCTATTATG | ACGTTGGATGGACCACTTCAGCATTCTAGG | GTACAATAACGTTAGCCC |
| ABCB1 | rs4728709 | ACGTTGGATGCCTAGCTCCTCTATTTAGCC | ACGTTGGATGATTGAGGTGAGTTGCATTCC | cAGCCCATCTGAGTCCA |
| RGS5 | rs2815272 | ACGTTGGATGAACAAGCAAAGCTAGTGCTG | ACGTTGGATGCACCACTTTTGTCCACCAAC | AGAAGCTAGTGCTGCTATTCAA |
| ADCY5 | rs4677889 | ACGTTGGATGAGGCAGAGAAGCAGACCTTG | ACGTTGGATGCACCCCCTGCTAGGATATAG | AGGATATAGGAGAGCACT |
| ADRA2A | rs12246561 | ACGTTGGATGGGGAGACAAAGGTACTGAAG | ACGTTGGATGTTGGTGCTGCTGCTGTTCAT | ACGTTGGATGGGGAGACAAAGGTACTGAAG |
| ADRA2A | rs1800038 | ACGTTGGATGGCTGCCAAGGCGTCGCGCT | ACGTTGGATGACACCACGAACACTCCGATG | TCGCGCTGGCGCGGG |
| ADRA2A | rs1800544 | ACGTTGGATGCCGTTGCGTTCTGCTCCGT | ACGTTGGATGAGACTTAAAGAGGGAGCCCG | TTGGCCATGCAGCTC |
| ADRA2A | rs2484516 | ACGTTGGATGTTCATGCGGCCCCCACACT | ACGTTGGATGGAGACTTCCAAAGTTGTGCG | TGTTCGCCGCCGCCGCCGTCCC |
| ADRA2A | rs553668 | ACGTTGGATGATTCCCCTTCCATTCCCAAC | ACGTTGGATGTAGTGTATATTTACAGCGGG | AGTTCCCAACTCTCTCTCTCTTTTT |
| ADRA2A | rs3750625 | ACGTTGGATGATTCCCCTTCCATTCCCAAC | ACGTTGGATGTAGTGTATATTTACAGCGGG | CTGGGGAGGGCAGGCAG |
| ADRA2A | rs11195419 | ACGTTGGATGCTACAAGGGAAGCTTCTTGC | ACGTTGGATGTAGGAAGATGGCTCCAGGTC | GGCTCCAGGTCAAGAGTG |
| ADRA2A | rs1800545 | ACGTTGGATGCTCCCGCCGCTTAGAAATAA | ACGTTGGATGTCGCGCTTCAGACGCTCTC | ggggTCGGAGCAAGAAGGC |
| ADRA2A | rs41286894 | ACGTTGGATGACACAGCTGTCTGGTTCAGG | ACGTTGGATGGAAAACGACGACATAGAGGG | ccgtGCTTGCATTGCAAAGG |
| ADRA2A | rs13306145 | ACGTTGGATGAGTAAATGAGCCTTTCTGCC | ACGTTGGATGGGCAAACAGTGCATCAGAGA | CCTCCCATCAGCCCTGTGTATAAA |
| ADRA2A | rs12772798 | ACGTTGGATGAGACAATTTAAGGCCTGCAC | ACGTTGGATGAAAATCCACTGTCATTAGC | GGGGTCCACTGTCATTAGCAACATG |
| ADRA2B | rs4907299 | ACGTTGGATGGAAAAGTGGAAGGCTGGCTC | ACGTTGGATGGCTTGTGGTGTTTTCGTTTC | TTCGGGCTCCGTGCTCTTTGT |
| ADRA2B | rs7604842 | ACGTTGGATGGTTTTGTGATTTCAAGACC | ACGTTGGATGTCACAACCTTGCTGAGAATG | CCACACGAAATTGAGACTTAAAGTG |
| ADRA2B | rs9333567 | ACGTTGGATGCGTTGCCGAAGATGGTAAAG | ACGTTGGATGATGGACCACCAGGACCCCTA | AGGTTATGGCCGCCGCTATGGCCGC |
| ADRA2B | rs3813662 | ACGTTGGATGAGGTGGTGGTAAACACAGAG | ACGTTGGATGAAAGCTTTGAGCTCTGTGGG | AATCACATTTTTGGTTCTCT |
| ADRA2C | rs13109333 | CTCTCTGCCCAGGCCTGGT | ACGTTGGATGGAGAACATGGCCAGTCAAGG | ACGTTGGATGACAAGCTGGGCCAACAGTTC |
| ADRA2C | rs6846820 | ACGTTGGATGGCAGGCCCATCATCTGAAAT | ACGTTGGATGTCACCATGTTTGTCACCAGC | TTCTTGGGTGTGGAGGCCGAGCC |
| ADRA2C | rs76337672 | ACGTTGGATGTAAATGGGCAAGCAAGGAGC | ACGTTGGATGAGAAGTCAGCCCTTGGTCAG | GGGGAGTGGTAGTGT |
| ADRA2C | rs7434630 | ACGTTGGATGGAGAAGCGCTTCACCTTTGT | ACGTTGGATGGCTGTAGCTGAAGAAGAAGG | ggggACCAGCAGAGCACGAACAC |
| ALB | rs7670092 | ACGTTGGATGACTTTGTGCCCATTGATTAG | ACGTTGGATGTATAATGCTGGTTGCCAGTG | CCCATTGATTAGTAACCCCT |
| CACNA2D2 | rs2236957 | ACGTTGGATGTCCTGTCCCCTCATCAGTC | ACGTTGGATGACTGTCACACTCAACGACCA | CCCCGTGCCCACACA |
| CACNB2 | rs7069292 | ACGTTGGATGCCGCCCAGCCTCCTCAGTT | ACGTTGGATGTCAGTGCTGTGCTAAGGTGC | GCCTCCTCAGTTCTTATA |
| CACNB2 | rs12258967 | ACGTTGGATGGTCATCGGGGAGAAAATAAC | ACGTTGGATGGTCTCGTATCCTGTGAGATG | cAAATAACTTCTCTACCCTCTT |
| CACNB2 | rs2228645 | ACGTTGGATGGACATTTGACTCGGAAACCC | ACGTTGGATGACGTGGTCATGGGAATAATC | acTAATCTTCCTTTGGCTCTAC |
| CACNB2 | rs2482100 | ACGTTGGATGGCAAATGCAGAGGCAACAAG | ACGTTGGATGATCCACTGGTGCTTTGATCC | gggcAACAACAGACTGTACCTT |
| CACNB2 | rs35803482 | ACGTTGGATGGAATGACTCTATTTCGGTGG | ACGTTGGATGTTCTGCACAAGCTATCACTG | GACACTTAGTTTTTGTAAAACAC |
| CACNB2 | rs61839258 | ACGTTGGATGCTTTTAACAATTTGAGCGCAG | ACGTTGGATGGCATTCAAAAGCCGAGCATC | aattCATCTCATAACCCTAAACATT |
| CACNB2 | rs16916932 | ACGTTGGATGATGGTAGTAAGTGAGCATAC | ACGTTGGATGCTGAAAATGTTAAGGCAGGTC | GTGAGCATACATTAATCTGAAAAAG |
| CACNB2 | rs11014166 | ACGTTGGATGTTGTGATCAGCTGTGACAGG | ACGTTGGATGTAGAGATAGTAGTGCATTCC | CAGGCTATTAGTATTGTTATTTTCCT |
| CACNB2 | rs10764319 | ACGTTGGATGACACTAAGACTTTTCACCCC | ACGTTGGATGAAAGTGCTGGGATCACAGGG | cacgTTTCACCCCTATAGAAAAAC |
| CALM2 | rs815815 | ACGTTGGATGGGAACACATTTTCTCTAGGG | ACGTTGGATGACAAAAATTGTTGAGCAGGG | tgCAAGAAGGAATCCAGAAAG |
| CAMK1D | rs7100726 | ACGTTGGATGCCTGGCCTGACTCTTTCATT | ACGTTGGATGTGTGCCTTGCAGAATCATAG | ggTAGTTGTGGTGTCTCAAAC |
| GNB3 | rs2071057 | ACGTTGGATGTGTGGTTGGCTCTACCCTTC | ACGTTGGATGAGGCTCTGCTGCCAGAGGA | ggCCCCAACCCCCCGCC |
| GNB3 | rs5446 | ACGTTGGATGTGGGTGGTATAGGGCGTTTG | ACGTTGGATGAATAAGAAGAGGGCCAGGAC | gggtgGGGCCAGGACCCTAGT |
| HNF4A | rs745975 | ACGTTGGATGCCCTTAGATGCCTGACATTC | ACGTTGGATGGAGGTTGGTGCCTTCTGATG | TGGGGACGTGTCTGC |
| HNF4A | rs6130615 | ACGTTGGATGAACACAGCAGTTCTGCAGAG | ACGTTGGATGAGCCCCAAGCCTCATTACTC | CACCTCATTACTCTCACCC |
| ITPR1 | rs6796205 | ACGTTGGATGATCACGTAGCTAGAAGTGGC | ACGTTGGATGGGCAGTTTAGAGTAGCTGTT | GCTGTTAAGAGGAGGATTT |
| MAPK13 | rs2859136 | ACGTTGGATGCTAATCTCATCAGCTCCCAC | ACGTTGGATGGCAGAATGAACCTTACGGTG | cGTGTCCCACACTGAC |
| MAPK14 | rs851006 | ACGTTGGATGGTCAGTGTTACCACAATGTC | ACGTTGGATGCACTCTAATTCAGCTTCTAC | CAGCTTCTACATATCATAAAGG |
| MAPKAPK3 | rs11130254 | ACGTTGGATGTCACTATTCCCGACCCAAAG | ACGTTGGATGGGCTTTTGTCACAGTGACAT | TCCCACCTTAAGTTCAGA |
| NR1I2 | rs1523127 | ACGTTGGATGAGCCAAGTGTTCACAGTGAG | ACGTTGGATGAGGATCGATCAAGGAGTAGC | tAGGAGCCGCTGCCTT |
| NR1I2 | rs3814057 | ACGTTGGATGTAGCCACTTGTGAGTAAAA | ACGTTGGATGAGGGCTACATTTCCCAAAAC | ACATTTCCCAAAACTAGTTC |
| NR1I3 | rs2307418 | ACGTTGGATGTTTCACCAACCCCTTCCTGC | ACGTTGGATGAAAGCTACATCAAGGGCCAG | GTGGCCTCCAAGCCC |
| NR1I3 | rs2307424 | ACGTTGGATGACTGAAGTGTTTGCCTCCTG | ACGTTGGATGTCATGGTACTGCAAGTCATC | ATGGCACTCACCGGAAGAC |
| NR1I3 | rs4073054 | ACGTTGGATGCTGAAACGATGTGAGACAGG | ACGTTGGATGTTACTGTCCTTTCCTTAGGG | CCTTAGGGAATTCAGGTATC |
| NSUN6 | rs75967699 | ACGTTGGATGTTCAGTTGGCCTGACAACAC | ACGTTGGATGGGGATGGATGCTCAGAAATG | ggagACACTTTGGTTAAAAAAAAAAAA |
| PRKCB | rs1015408 | ACGTTGGATGCATTTCTGCTCTTGTTACCG | ACGTTGGATGTTTCCTACTGTCCAGATGCC | ACGATTCTTATCTGCACTTTAC |
| PRKCB | rs11074583 | ACGTTGGATGAATGTCCTAAAGACCTCATC | ACGTTGGATGCCACCTGCATACAAATCTTC | TTCCTCTGCAACTCTGGCTA |
| PRKCB | rs114556512 | ACGTTGGATGCCTTTGGTTGCAAGTAACAT | ACGTTGGATGCCTTCACATCTCTGTCCATC | AGTCCATCCCTCCCCC |
| PRKCB | rs3785380 | ACGTTGGATGATGTGCTTTTAGACGGTCTC | ACGTTGGATGCTACAACACTGAGGTAGCAC | ATGGTTGCCCTTTGACCC |
| PRKCB | rs1976194 | ACGTTGGATGTGGTTTCACCCTTGGGTAAC | ACGTTGGATGTGGGACACCTATGAACCATC | TGGGTAACTCAGTACAGGTATGT |
| PRKCB | rs2051684 | ACGTTGGATGCTCTGCAAGACTCTAGAACC | ACGTTGGATGCAGGAATGTGAAGTTAAGGC | CCCGGAAGCCTTTGCTAAC |
| PRKCB | rs75964872 | ACGTTGGATGAGGAGAGCACCGTGCGCTT | ACGTTGGATGTCTTGACCTCATGCACGTTC | GCACGTTCTTCTGCC |
| PRKCB | rs11074601 | ACGTTGGATGCTGAGGTCCAGAGAAAGGTT | ACGTTGGATGGAAGGGAGATCTACCAGAAC | GGATCTGTGTGTGACCTG |
| PRKCB | rs16972959 | ACGTTGGATGCCAATAGTTAGAATGGTACT | ACGTTGGATGAAGTCAAGTAGATCCATTC | ccacAAATATGTGCTGAGTGTCTA |
| PRKCB | rs198188 | ACGTTGGATGCCTCTTTCCTGTATTCCCAC | ACGTTGGATGAGGAACTGTCAGAATTCGCC | CCCTCCCACAGCCATGTAC |
| PRKCB | rs3729904 | ACGTTGGATGTTCTCTCGAAGCTGATGACC | ACGTTGGATGTACCGGAAAAATGCATGCTC | tgccTGCTCTTTGATATCACGTTC |
| PRKCB | rs429342 | ACGTTGGATGTTAACAGAGCAGGGCCTTTC | ACGTTGGATGCCATCTAGATCCACAAGCTG | GCCTTTCTCTGACAGCAAG |
| PRKCB | rs7202459 | ACGTTGGATGCCAATAGTTAGAATGGTACT | ACGTTGGATGAAGTCAAGTAGATCCATTC | ccacAAATATGTGCTGAGTGTCTA |
| PRKCB | rs8054767 | ACGTTGGATGTGCAGTATCTCACTGAACTC | ACGTTGGATGGTTTTCGTTTCCTTGTGTGG | CCTAGGAATTAAGCATTATTATTATT |
| PRKCB | rs2239339 | ACGTTGGATGTCACATAGACTTGGCAAGAG | ACGTTGGATGTTGCTTCTTTGTGGCTGCTC | AGGAGGGAACTCCATA |
| PRKCB | rs9922316 | ACGTTGGATGATCGGTCTCATTTTGAGGGC | ACGTTGGATGCTTTTCTGCCAATCTTCACC | CATCTTCACCTAGTTTTTGTTT |
| PRKCE | rs1143691 | ACGTTGGATGTCGTTCACGGTTCTATGCTG | ACGTTGGATGAGAGGCTACCTGTAGATGAC | TGTAGATGACTCCATGCTG |
| PRKCE | rs1464572 | ACGTTGGATGGTCAGATATGTCCTGGGAGC | ACGTTGGATGCTACAAGCCCTCTATGTCAC | GATGGCCGAGGCAATAGGATCA |
| PRKCE | rs1868389 | ACGTTGGATGCTATTTCCTACCTATTGGAG | ACGTTGGATGCTAACCTTGGCTAGGTTCTC | tccaTATTGGAGGTAGGCGGG |
| PRKCE | rs2711285 | ACGTTGGATGATTCCACTGGTGGAAACAAG | ACGTTGGATGCTTGGGTACTGATACTCTGC | cgGGTGGAAACAAGACTTCA |
| PRKCE | rs10191412 | ACGTTGGATGGAACCTATGGGGATAGAAAG | ACGTTGGATGCTCCTTCACAGTCAAGTACC | cctTCAAGTACCCAACCAAAG |
| PRKCE | rs13432276 | ACGTTGGATGCTTCGCTAACCTTCCTCTAC | ACGTTGGATGAATGCCAGGACATGGGAACG | cctcgACAACTGATCTGGTCAGTTT |
| PRKCE | rs4953260 | ACGTTGGATGCTTGTAGAACCGTAACTGTC | ACGTTGGATGTTGCCTGTGCTTATGAGGAG | gtagGAGGAGAGGGGAATGGGG |
| PRKCE | rs4953268 | ACGTTGGATGTTAACTACAAGAGGCCCAGC | ACGTTGGATGCCACCTCAAATCCAGCTAAC | aatttAAATAATTGGGTGAGACAAAA |
| PRKCE | rs921183 | ACGTTGGATGGGACCTAAAATGCTTTAGGC | ACGTTGGATGGCTGGAAACTGAAGCAGTTC | ggGAAGCAGTTCCAAATTTTC |
| PRKCE | rs628877 | ACGTTGGATGGAAGGGCTATTTAGAGGGAG | ACGTTGGATGCAGAGATGGGCAAAATCGAG | ctCCTCTTATACCCCCATCC |
| PRKCE | rs6720975 | ACGTTGGATGTGAATCCCTAGGAGTGTCAG | ACGTTGGATGATCAAACGCTAGACAGCAAG | TCTCCCTCTTCCCAA |
| PRKCE | rs10189339 | ACGTTGGATGGCCTACCAAAGTGCTGGAAT | ACGTTGGATGTTAACACCGTCGCCTGCTTC | CCCGCCATTGTTAGT |
| RGS20 | rs10435634 | ACGTTGGATGGCATTATACTTACCAGTTG | ACGTTGGATGTGAGCATTGACATGACACTC | agTACCAGTTGAACATCCCAA |
| RGS5 | rs6691456 | ACGTTGGATGAACAACAGATGCTGGCAAGG | ACGTTGGATGGCTTTCCACAGTGGCTAAAA | ggTTCCATCAACAGCCTT |
|  | rs1017437 | ACGTTGGATGCCCCAAGTTCCCAACTTATC | ACGTTGGATGGCTTTCAAGGACTAATCAG | cTTTGTTTTTCACCGATCTTTTA |
|  | rs3767489 | ACGTTGGATGGTCTCCTGACTTTGATCAGC | ACGTTGGATGGCAGTTACAGGGAAGATCAT | atATCATTCATTCTCAGATATCTTGT |
